# Deformation Behavior and Accuracy Control in Gas-Assisted Diaphragm Forming of Composites Using Multi-Point Flexible Die

**DOI:** 10.3390/polym18050551

**Published:** 2026-02-25

**Authors:** Deyu Yue, Ruixiang Luo, Yuan Li, Zhe Wang, Hexuan Shi, Huifeng Mei, Xianglin Chen, Long Cao, Junhang Xu, Yunzheng Han, Qigang Han

**Affiliations:** 1 Key Laboratory of Automobile Materials, Ministry of Education, School of Materials Science and Engineering, Jilin University, Changchun 130022, China; 2 Institute of Structured and Architected Materials, Liaoning Academy of Materials, Shenyang 110167, China; 3 Weihai Institute for Bionics, Jilin University, Weihai 264207, China

**Keywords:** gas-assisted diaphragm forming, multi-point flexible die, processing parameters, dimple defects, geometrical accuracy

## Abstract

Multi-point flexible die (MPFD) exhibits broad application potentials for efficient and controllable forming of curved sheets due to its rapid reconfigurability. Nevertheless, the relatively poor surface accuracy and geometrical accuracy of the fiber-reinforced composite components formed by MPFDs limit the widespread application of this technology. In this study, a novel gas-assisted diaphragm forming (GADF) process based on MPFDs for curved basalt fiber/epoxy resin composite sheets was proposed. The precise control of temperature, pressure and MPFD configuration in the process was realized and verified. The effects of different process parameter configurations on dimple defects and geometrical accuracy were analyzed, and the mechanism of defect generation was investigated. A response surface-based forming accuracy prediction model was developed to analyze the influence of component structural parameters on geometrical accuracy. Based on the predictive model, compensation reconfiguration of MPFDs was carried out to achieve high-accuracy sheet forming. Results demonstrated that increasing pressure exacerbated the dimple while reducing shape accuracy. A moderate temperature (120 °C) was proved optimal for component forming, as both excessively low and high temperatures aggravated dimple and induced geometrical errors. Increasing interpolator thickness effectively reduced dimple defects, but excessive thickness adversely affected component geometrical accuracy. Considering both dimple suppression and geometrical accuracy, the optimal process parameters were determined to be 5 kPa, 120 °C, and 2 mm of interpolator thickness. Through MPFD modification based on the response surface model, the geometrical accuracy of the formed components was improved by 38.85%, achieving high-quality forming of the curved composite sheets.

## 1. Introduction

Curved sheets have been widely used in the field of aerospace, rail transportation, and automotive industry due to their superior dynamic performance [1]. The materials for curved sheets can be categorized into metals, polymers and composites, among which fiber-reinforced composites are increasingly adopted due to their lightweight characteristics and excellent mechanical properties [2]. Fiber-reinforced composite curved sheets typically feature complex geometries and high dimensional accuracy [3]. With accelerating design iteration speeds, fiber-reinforced composite curved sheets now require small-batch, multi-variety production capabilities. However, conventional solid dies are inherently limited by their one-die-one-part configuration. The forming methods based on solid dies suffer from prolonged production cycles and prohibitively high manufacturing costs, which significantly constrain broader applications of fiber-reinforced composite curved sheets.

Multi-point flexible dies (MPFDs) discretize continuous surfaces via height-adjustable pin arrays. This reconfigurable mechanism generates diverse surface topographies, enabling multi-surface capability with a single die. Nakajima [4] pioneered the concept of reconfigurable MPFD for sheet metal forming in 1969. Subsequent technological advancements have enabled the integration of MPFDs with various forming techniques for different materials. Among them, press forming, as one of the predominant sheet-forming methods, has been deeply combined with MPFDs [5,6,7,8,9,10,11]. Zhang et al. [12,13] developed a sandwich-forming MPFD by replacing the multi-point upper die with an elastomeric rubber tool. This modification reduced the overall cost and improved the forming quality compared to press-forming MPFD. For large, curved metal sheets in aerospace, stretch forming is commonly used. Stretch-forming MPFD replaces traditional solid dies with multi-point dies, enabling rapid forming of aircraft skins [14,15,16]. Incremental forming is a sheet manufacturing process based on accumulated localized deformation. This technique utilizes a hemispherical punch to progressively deform metal or composite sheets through sequential toolpath movements [17,18,19]. However, incremental forming has shortcomings in terms of geometric accuracy and uniformity of thickness distribution. The formability and accuracy of incremental forming was enhanced by researchers through effectively supporting sheets with MPFDs [20,21,22].

The MPFD-based forming processes mentioned above show limited suitability for fiber-reinforced composite curved sheets, while the diaphragm-forming MPFD hybrid system offers a promising solution through either vacuum pressure operation or assisted external pressure mode. The pressure sequentially deformed the diaphragm to conform the sheet to the MPFD surface. Diaphragm forming allows easier heating of sheets and more controllable pressure application to the diaphragm. Therefore, this process is primarily used for forming polymers [23,24,25] and fiber-reinforced composites [26,27]. Walczyk et al. [26] proposed a forming method based on MPFD and diaphragm heating, achieving automated manufacturing of composite components through vacuum assistance combined with an interpolation layer. Wimmer et al. [27] addressed forming challenges for convex and transitional structures of fiber-reinforced composite using a hexagonal dense pin array, with experiments demonstrating that a 20 mm thick interpolator significantly eliminates dimple defects, providing a novel approach for low-cost manufacturing of composite sheets. However, the sheet formed by the diaphragm-forming MPFD currently still has instability, and its process characteristics need to be further explored.

Forming accuracy is critical in composite material forming using MPFDs. Forming accuracy can be categorized into surface accuracy based on dimple defects and geometrical accuracy affected by factors like curing deformation. While discrete MPFDs can adapt to forming sheets with varying curvatures, it introduces surface discontinuities that lead to dimple defects [28]. The application of interpolators is a conventional method for eliminating dimples [29]. Abebe et al. [30] employed multi-objective optimization of stamping pressure, stamping speed, and interpolator thickness to mitigate dimple defects. Qu et al. [8] investigated the materials and configurations of interpolators, demonstrating that steel-backed polyurethane pads effectively improve blank stress–strain distribution and suppress dimple. On the other hand, springback deformation in metal sheets [31] and curing deformation in polymer or fiber-reinforced composite sheets [32] are primary factors affecting the geometrical accuracy of curved sheets. Leveraging the flexible adjustability of MPFD surfaces, rapid reconfiguration of MPFDs to compensate for errors presents an effective approach for enhancing geometrical accuracy [33].

Although previous researchers have made some attempts to apply MPFD to composite sheets forming, a reliable equipment system capable of stably producing fiber-reinforced composites has not yet been established. In this work, we proposed an innovative GADF-MPFD process that integrated gas-assisted diaphragm-forming technology with multi-point dies, fully leveraging the controllable process parameters of GADF and the flexibility of MPFD. The primary objective of this study was to develop a series of stable process parameters to ensure high-accuracy forming of composite sheets. Accordingly, we first determined the configuration accuracy of the MPFD. Comparative analysis of sheet profiles under varying process conditions enabled the identification of root causes for dimple defects, with subsequent parameter optimization achieving defect-free sheet forming. Building upon this foundation, we systematically investigated the effects of various process and structural parameters on geometrical accuracy. Furthermore, we established a reliable predictive model for curing deformation based on structural parameters. The predictive model enabled the production of high-accuracy curved composite sheets by utilizing the reconfigurable characteristics of MPFDs. The developed GADF-MPFD demonstrated significant improvements in accuracy control compared to conventional forming methods.

## 2. Theoretical Analysis of the Forming Process

**Figure 1:**
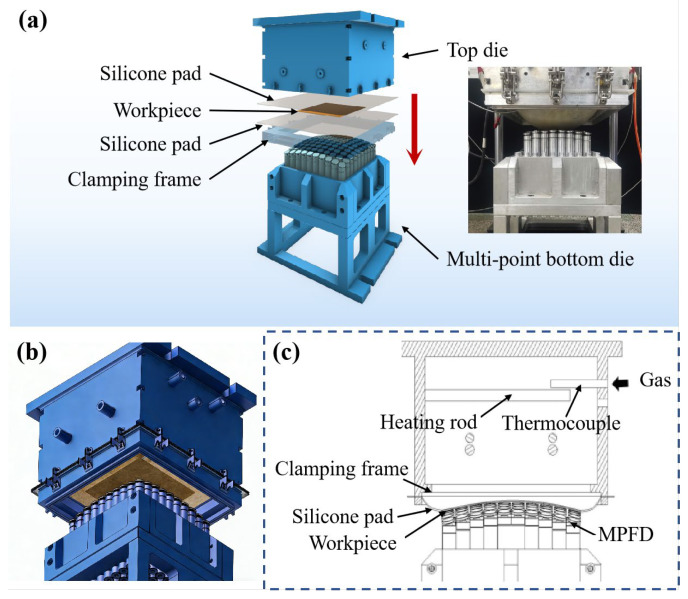
(**a**) The forming module, (**b**) assembly view, and (**c**) cross-sectional view of the GADF-MPFD equipment.

The GADF-MPFD equipment could be divided into three main modules: the forming module, the heating temperature controller, and the gas pressure controller. The forming module comprises a top die with a gas chamber, a clamping frame that holds the silicone pad and the workpiece, and a reconfigurable multi-point bottom die, as shown in Figure 1: (a) the forming module, (b) assembly view, and (c) cross-sectional view of the GADF-MPFD equipment. The clamping frame is securely fastened to the top die via uniformly distributed clasps around its perimeter. The side wall of the gas chamber in the top die is designed with several air inlets. Gas is introduced through these inlets to apply pressure to the diaphragm. Inside the gas chamber, four heating rods are arranged, and the temperature is monitored through corresponding thermocouples. The multi-point bottom die is positioned at the base of the forming module. It allows for the flexible reconfiguration of its pins, thereby enabling the formation of fiber-reinforced composite sheets with diverse curvatures.

The silicone pad serves three core functions: (1) uniformly transmitting gas pressure to avoid local stress concentration; (2) isolating and protecting the composite surface from scratches; (3) assisting in uniform heat conduction. It also acts as a release layer for easy demolding.

The contact portion of the mold is the key part affecting the forming quality, which mainly includes the contact surface of the upper die gas chamber, the swinging head of the multi-point bottom die and the silicone pad. The contact surface of the upper die gas chamber is made of silicone pad with a smooth surface, and the contact area is 250 mm × 250 mm. The multi-point bottom die adopts a design based on swinging pins, and the swinging head is made of 6065 aluminum alloy with a spherical structure. The contact mode between the swinging head and the silicone pad is surface contact, which effectively reduces the stress concentration during the forming process. The silicone pad has a low friction coefficient with the prepreg. Moderate friction prevents excessive interlayer slip of the prepreg without hindering resin flow, which is beneficial for improving forming quality. Pre-experiments confirm no adverse effects from this friction.

The forming process of fiber-reinforced composites by GADF-MPFD equipment can be divided into the following steps:

(1) Cutting: The prepreg is retrieved from the refrigerator and cut into the desired shape according to the designed structure.
(2) Reconfiguring: The height of the pins for the multi-point bottom die is calculated based on the target surface geometry. The pin heights of the multi-point bottom die are adjusted using a robot to complete the reconfiguration of the die surface.
(3) Clamping: A release agent is sprayed between two layers of silicone pads. The cut prepreg workpiece is arranged within the silicone pads. The silicone pads and the workpiece are secured in a sandwich configuration beneath the top die using a clamping frame and pressed tightly against the multi-point bottom die.
(4) Heating and Pressurization: The temperature controller is activated to begin heating. As the temperature rises, the resin gradually softens. The gas chamber of the top die is inflated to cause the silicone pads to bulge. The pressure controller is adjusted, and the pressure gauge is observed to determine the forming pressure, ensuring that the resin deforms and flows under the applied pressure.
(5) Curing: After heating for an appropriate duration, the temperature controller is turned off, and the assembly is subjected to ambient cooling (natural cooling in the air at 25 ± 2 °C) at room temperature. The cooling rate is controlled at about 2–3 °C/min. During the cooling process, the pressure in the gas chamber is maintained as constant to ensure that the fiber-reinforced composite remains in close contact with the MPFD. Once the temperature of the workpiece drops to room temperature, the silicone pads are disassembled, and the mold is opened to obtain the curved fiber-reinforced composite workpiece.

### 2.1. Temperature Controller

Temperature is a critical processing parameter in the thermoforming of fiber-reinforced composites. During the forming process, the resin matrix must be subjected to heat and pressure to initiate the curing reaction. If the temperature is too low, the material will not reach the necessary curing temperature, while excessively high temperatures can cause thermal degradation, carbonization or decomposition of the resin matrix [34], and also lead to a series of forming quality problems of composite parts. When the forming temperature exceeds the target temperature, the fluidity of the epoxy resin matrix will increase sharply, leading to resin loss during the forming process and uneven fiber–resin ratio of the parts, which affects the uniformity of the material performance. Meanwhile, the excessive temperature will cause a significant increase in the thermal stress of the composite material during the cooling stage, leading to serious curing deformation and a sharp decrease in the geometrical accuracy of the parts. In addition, the excessive temperature will reduce the interface bonding force between basalt fiber and epoxy resin, and even cause interface debonding, which seriously reduces the interlayer shear strength and other mechanical properties of the composite parts.

The temperature controller comprises a relay, a microcomputer, a power supply, four heating rods, and multiple thermocouples. It enables the feedback of temperature measurement data from the thermocouples and controls the switching of the heating rod circuits. The temperature controller has four channels, which display and control the temperatures of four heating rods, respectively.

Heat penetrates the silicone pad and is transferred to the fiber-reinforced composite. During the heat transfer process, heat losses will occur. Therefore, it is necessary to calculate an appropriate input temperature to adjust the forming temperature. For a stable temperature field, heat conduction follows Fourier’s law, which is expressed as:

(1) $$Q=-\lambda A\frac{\partial t}{\partial n}$$

where *Q* is the heat flux, which refers to the amount of heat passing through the silicone pad per unit time, *λ* is the thermal conductivity, which is set as 0.57, *A* is the area of the silicone pad, and $\frac{\partial t}{\partial n}$ is the rate of change in temperature along the *n* direction.

For heat conduction through a silicone pad, the heat flux after integration can be expressed as:

(2) $$Q_{s}=-\lambda A\frac{t_{2}-t_{1}}{b}$$

where *Q_s_* represents the total heat flux passing through the silicone pad, *b* is the thickness of the silicone pad, and *t*_1_ and *t*_2_ are the temperatures on either side of the silicone pad, respectively.

Aluminosilicate insulation materials are laid on the top surface and the four side surfaces of the gas chamber. The heat transfer model for the top surface and the four side surfaces is simplified to a double-layer flat wall with stable heat conduction. After the heating reaches a stable state and the aluminosilicate insulation layer is in complete contact with the aluminum alloy outer frame. By integrating the Fourier equation, the heat flux dissipated during the operation of the equipment can be obtained:

(3) $$Q_{d}=-\frac{t_{3}-t_{1}}{\frac{b_{1}}{\lambda_{1}A_{1}}+\frac{b_{2}}{\lambda_{2}A_{2}}}$$

where *Q_d_* is the heat flux dissipated through the upper surface or side surface, *t*_3_ is the temperature value of the outer wall of the aluminum alloy; *b*_1_ and *b*_2_ are the thicknesses of the aluminosilicate plate and the aluminum alloy plate respectively, *λ*_1_ and *λ*_2_ are the thermal conductivities of the aluminosilicate and the aluminum alloy plate respectively, and *A*_1_ and *A*_2_ are the areas of the aluminosilicate plate and the aluminum alloy plate respectively.

The conventional curing temperature for epoxy resin-based composites is typically set at 120 °C [35]. Therefore, the target heating temperature of the equipment in this study was established at three levels: 110, 120 and 130 °C. The total heat calculated by the above formulas was input into the temperature controller. The temperature distribution of the silicone pad was accurately monitored through the combination of real-time temperature regulation and thermal imaging technology. The thermal images captured were analyzed in depth using the HIKMICRO Analyzer V2.0 software accompanying the thermal imager, with a focus on examining the temperature gradient changes along the diagonal line L and the temperature distribution characteristics within the molding area R.

**Figure 2:**
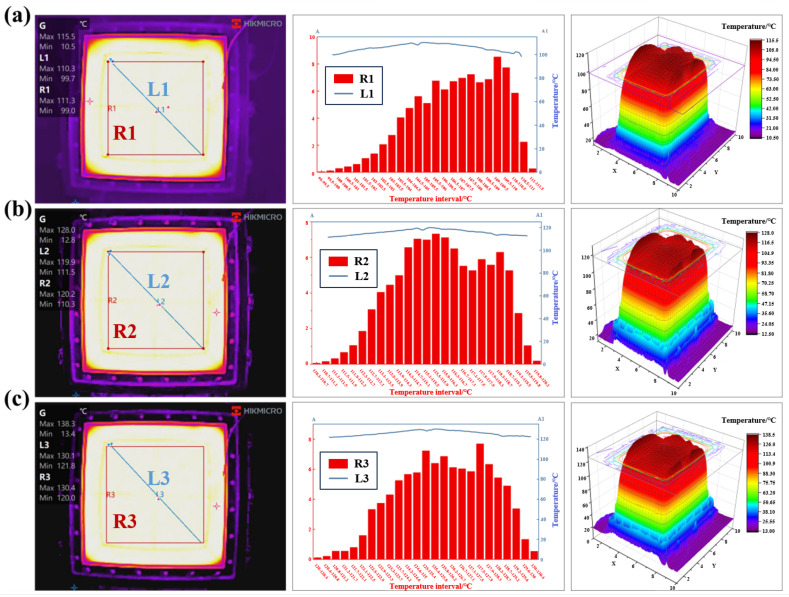
Thermal images and the analysis data at different target heating conditions: (**a**) 110 °C, (**b**) 120 °C, (**c**) 130 °C.

The analytical results of the thermal images are presented in Figure 2. Defined R1–R3 as the molding areas for target temperatures 110/120/130 °C, and L1–L3 as their corresponding diagonals in the caption and text. Within the thermal images, white regions denote elevated temperatures while black areas indicate lower thermal values. The silicone pad surfaces under all three temperature conditions exhibit whitish coloration with homogeneous distribution, demonstrating effective thermal uniformity of the GADF-MPFD across the designated temperature ranges. At the 110 °C heating condition, the maximum temperature within the molding area R1 reaches 111.3 °C, with a minimum of 99.0 °C and an average of 106.8 °C, yielding a 3.2 °C deviation from the target temperature. The temperature distribution chart of R1 reveals predominant temperature concentrations in the range of 103.5–110.5 °C, confirming satisfactory uniformity across the working area. Along the diagonal line L1, temperature variations follow a low-high-low transitional pattern with minor fluctuations. Similar thermal distribution patterns are observed at the heating conditions of 120 °C and 130 °C, with critical performance parameters systematically summarized in Table 1. The stable temperature uniformity verifies the temperature controller’s reliability, guarantees uniform composite curing, and provides a valid temperature control basis for subsequent process parameter optimization.

### 2.2. Pressure Controller

Forming pressure also plays a significant role in the forming process of fiber-reinforced composites. Conventional diaphragm forming typically employs atmospheric pressure as the forming pressure [36,37]. However, for a hot-press-forming equipment based on an MPFD, excessive pressure would lead to a unique dimple defect in the formed composite material sheets [38]. Therefore, the appropriate application of pressure is crucial for the forming accuracy of the equipment. In this study, three target pressure levels were established: 5, 10, and 15 kPa.

The pressure controller mainly consists of an air compressor, a precision pressure regulator, a resistive thin-film pressure sensor and a pressure gauge. The air compressor is connected to the precision pressure regulator via a silicone tube, which adjusts the input air pressure to the air chamber to the target pressure. The resistive thin-film pressure sensor is installed at the outlet of the precision pressure regulator, which collects the real-time pressure data in the gas chamber and transmits it to the pressure display system to realize the real-time monitoring of the forming pressure. The working principle of the GADF equipment involves inflating the air chamber to expand the silicone pads and the workpiece beneath it, causing them to conform to the MPFD. In order to maintain the pressure for the target time, the precision pressure regulator has an automatic pressure compensation function: when the pressure in the gas chamber decreases slightly due to slight air leakage and other factors during the forming process, the pressure regulator will automatically open the air intake valve to supplement the air pressure, so as to keep the pressure in the gas chamber stable at the target value.

**Figure 3:**
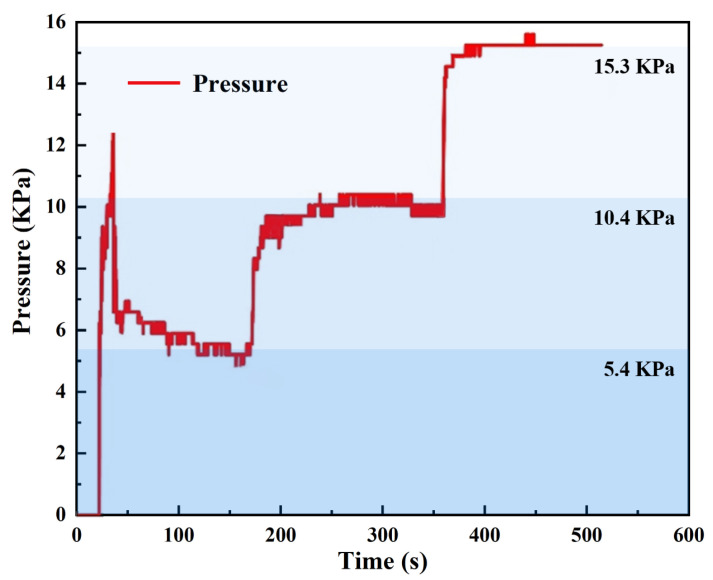
The pressure curve measured using a resistive thin-film pressure sensor.

A resistive thin-film pressure sensor is used to measure the actual forming pressure under different target pressure input conditions, as shown in Figure 3. It can be observed that the pressure exhibits significant fluctuations during the initial inflation stage, which is caused by the instability of the air pressure when the air compressor is first activated. After adjusting the target pressure to 5 kPa, the pressure eventually stabilizes around 5.4 kPa. After maintaining this pressure for 3 min, the target pressure is adjusted to 10 kPa, resulting in an actual measured pressure of 10.4 kPa. Finally, the target pressure is adjusted to 15 kPa, with the actual pressure measured at 15.3 kPa. It can be concluded that the difference between the measured pressure values and the target pressure values stabilizes within a relatively small range.

### 2.3. Theoretical Analysis of MPFD

Based on the movement characteristics, the pins in MPFD can be categorized into fixed pins and swinging pins [39]. The fixed pin features a spherical head with a fixed curvature, while the swinging pin is connected via a spherical hinge to an adaptive swinging head. By replacing the traditional fixed pin with a swinging pin, the contact interface between the pin and the workpiece transitions from point contact to surface contact. During the composite material forming process, swinging pins exhibit superior capability in achieving uniform stress distribution across the workpiece. Consequently, the MPFD in this study adopts a design based on swinging pins. The MPFD in this research employs 25 mm diameter pins arranged in a 10 × 10 square array, covering a forming area of 250 mm × 250 mm.

**Figure 4:**
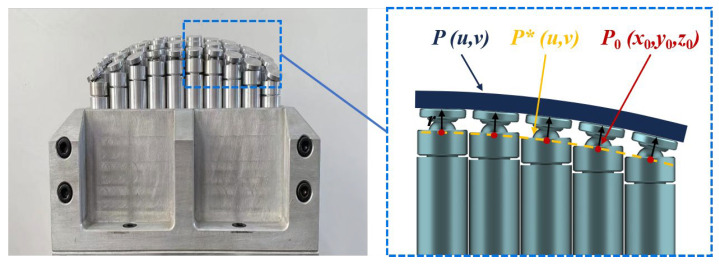
A schematic diagram of the pin height calculation.

To accurately guide the height of the pins of an MPFD, a method of constructing a NURBS fitting surface was employed. The schematic diagram for calculating the height of the pin is shown in Figure 4. The surface of a formed part can be represented as [6]:

(4) $$P(u,v)=\frac{\sum_{i=0}^{n}\sum_{j=0}^{m}B_{i,k}(u)B_{j,l}(v)W_{i,j}V_{i,j}}{\sum_{i=0}^{n}\sum_{j=0}^{m}B_{i,k}(u)B_{j,l}(v)W_{i,j}}$$

where *V_i_*_,*j*_ represents the control points of the NURBS surface, *n* and *m* define the number of control points in the *u* and *v* parameter directions, respectively, *W_i_*_,*j*_ represents the weights of the corresponding control points, and *B_i_*_,*k*_(*u*) and *B_j_*_,*l*_(*v*) are the B-spline basis functions in the *u* and *v* parameter directions, which characterize the surface through the control points.

The straight line *L* = *L*(*x*_0_,*y*_0_) corresponds to the forming pin, and the radius of the swinging head is *r*. The product of the normal vector of the surface and the radius of the swinging head determines the distance of the actual surface from the fitted surface:

(5) $$P^{*}(u,v)=P(u,v)\pm nr$$

where *n* is the unit normal vector of the surface at *V_i_,_j_*:

(6) $$n(u,v)=\frac{P_{u}(u,v)\times P_{v}(u,v)}{\left|P_{u}(u,v)\times P_{v}(u,v)\right|}$$

The new NURBS surface is solved by equidistant points, and the intersection of the newly constructed surface and the line *L* provides the height coordinate of the corresponding pendulum head:

(7) $$\left\{\begin{array}{c} x_{0}={\left.P(u,v)+rn\right|}_{x-x_{0}}\\ y_{0}={\left.P(u,v)+rn\right|}_{y-y_{0}} \end{array}\right.$$

## 3. Experimental Program

### 3.1. Materials and Preparation

Basalt, a mineral formed from volcanic eruptions, consistently exhibits excellent chemical stability, physical stability, and outstanding mechanical properties [39]. The basalt fiber prepreg was used to fabricate the sheets, with the prepreg composed of unidirectional basalt fiber reinforcements and an epoxy resin matrix. The basalt fibers (600 tex, area density = 200 g/m^2^) were provided by Zhejiang GBF Basalt Fiber Co., Ltd., Dongyang, China. The matrix material, bisphenol A epoxy resin (area density = 250 g/m^2^), was utilized as the bonding layer. Furthermore, the silicone interpolators were supplied by Hebei Hengdeli Sealing Material Co., Ltd., Hejian, China. The prepreg has a fiber–resin mass/area ratio of 4:5, and the cured composite has a fiber volume fraction of 32%.

**Figure 5:**
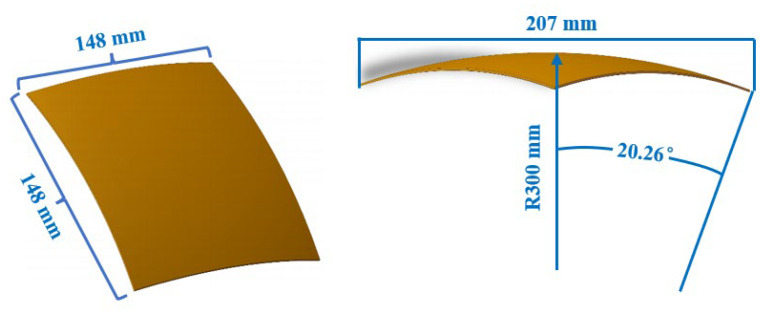
A schematic diagram of the spherical sheet.

The spherical sheet, as a commonly used and easily testable structure, was selected as the research subject. The size parameters of the target sheet are shown in Figure 5, the side length of the square composite blank is 150 mm, and the diagonal length of the effective spherical forming area after curing and forming is 207 mm. First, the basalt prepreg was cut into dimensions of 150 mm × 150 mm × 0.3 mm, and six layers of prepreg were stacked. Subsequently, by calibrating the pin height, the multi-point mold was adjusted to achieve a spherical shape with the target radius. In order to analyze the effects of pressure, temperature, and interpolator thickness on the forming quality of the sheet, eight sets of experiments were conducted, as shown in Table 2. Sets A1, A2, and A3 were employed to evaluate the pressure effects on formed components, Sets A2, A4, and A5 were used to assess the temperature effects, while Sets A2, A6, A7, and A8 were utilized to analyze the performance of the interpolator.

Due to the complex material composition of composite curved sheets, the structural parameters of the components also influence their forming accuracy. Based on optimized process parameters, a predictive model was developed using the Central Composite Design (CCD) method of Response Surface Methodology (RSM). RSM is an optimization method that integrates experimental design and mathematical modeling, which can effectively reduce the number of experiments and examine the interactions between influencing factors [40]. The range of forming layers is set to 8–12 layers, and the radius of the forming surface is set to 250–350 mm. Design-Expert 11.0 software is used to input the upper and lower limits of the design parameters of the forming samples. The specific experimental parameters are shown in Table 3.

### 3.2. Experimental Method

The surface accuracy and geometrical accuracy of curved sheets are the key indicators determining forming quality, while the configuration accuracy of MPFD serves as the prerequisite for sheet forming accuracy. The surface point cloud of MPFD was captured via portable optical 3D measuring instrument, NDI, Waterloo, Canada. Furthermore, the components were spray-coated with Xinmeida DPT-5 developer for surface coloration treatment followed by point cloud acquisition. The scanned data underwent point cloud processing and error analysis through Geomagic software 2020. Additionally, internal defects of components with/without dimples were analyzed using ultrasonic C-scan equipment from Beijing North Star Technology Co., Ltd., Beijing, China. The microstructure of cross-sections in dimpled components was examined with a KEYENCE VHX-6000 ultra-depth-of-field microscope, KEYENCE, Osaka, Japan.

## 4. Configuration Accuracy Control of MPFD

The configuration of the MPFD consists of multiple individual pins with varying heights, which are adjusted by a robot. During the robotic height adjustment process, mechanical and control errors cause deviations between the actual height of each pin and the target height. Inaccurate pin heights prevent a perfect formation of the target surface, limiting the widespread application of MPFD.

**Figure 6:**
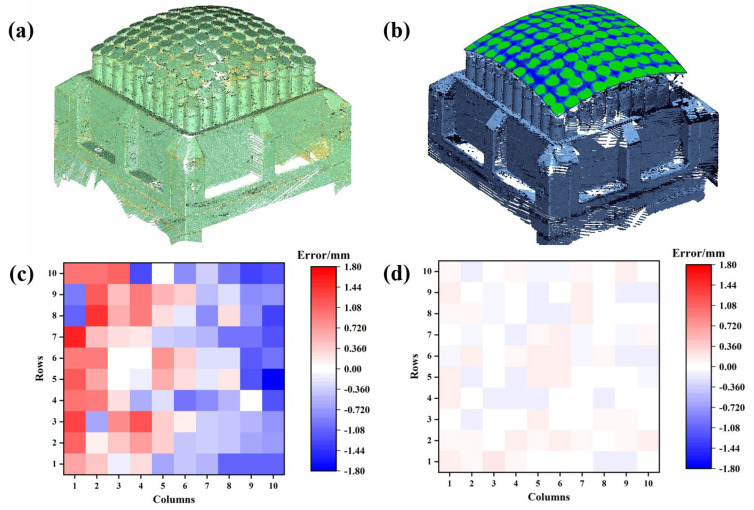
(**a**) Point cloud morphology of the MPFD, (**b**) the geometrical errors of MPFD and target surface, and the distribution of the pins’ height error (**c**) before and (**d**) after height compensation.

In this study, the point cloud of the MPFD after robotic adjustment was obtained using a portable optical 3D measuring instrument, as shown in Figure 6a. Figure 6b illustrates the data processing method, where an R300 target spherical surface was constructed on the point cloud to compare the deviation between the MPFD and the ideal surface. Reference points were selected on each pin and compared with the target spherical surface. The initial error distribution of the MPFD after the first adjustment is shown in Figure 6c, with pin height errors ranging from −1.792 to 1.522 mm and a standard deviation of 0.773. Based on the obtained errors, a height compensation calculation was performed, and the robot conducted a secondary adjustment to compensate for the deviations. The error distribution of the MPFD after height compensation is shown in Figure 6d, with pin height errors reduced to −0.136 to 0.165 mm and a standard deviation of 0.088. Through error acquisition and compensation, the consistency between the MPFD configuration and the target surface was significantly improved. In subsequent work, this method was consistently applied to ensure accuracy control of the MPFD.

## 5. Surface Accuracy Control and Dimple Suppression

In the GADF-MPFD process, the discrete arrangement of pins leads to surface discontinuities in the formed curvature. This results in stress concentration at the edges of pins, ultimately causing dimple defects on the surface of fiber-reinforced composite sheets. As a common defect in MPFD-based forming processes, dimples primarily occur at the pins and their peripheral regions. The formation of these dimples adversely affects both the surface accuracy and mechanical properties of the sheets. Therefore, effective dimple suppression is critical for ensuring product quality.

### 5.1. Effect of Process Parameters on Dimple Defects

**Figure 7:**
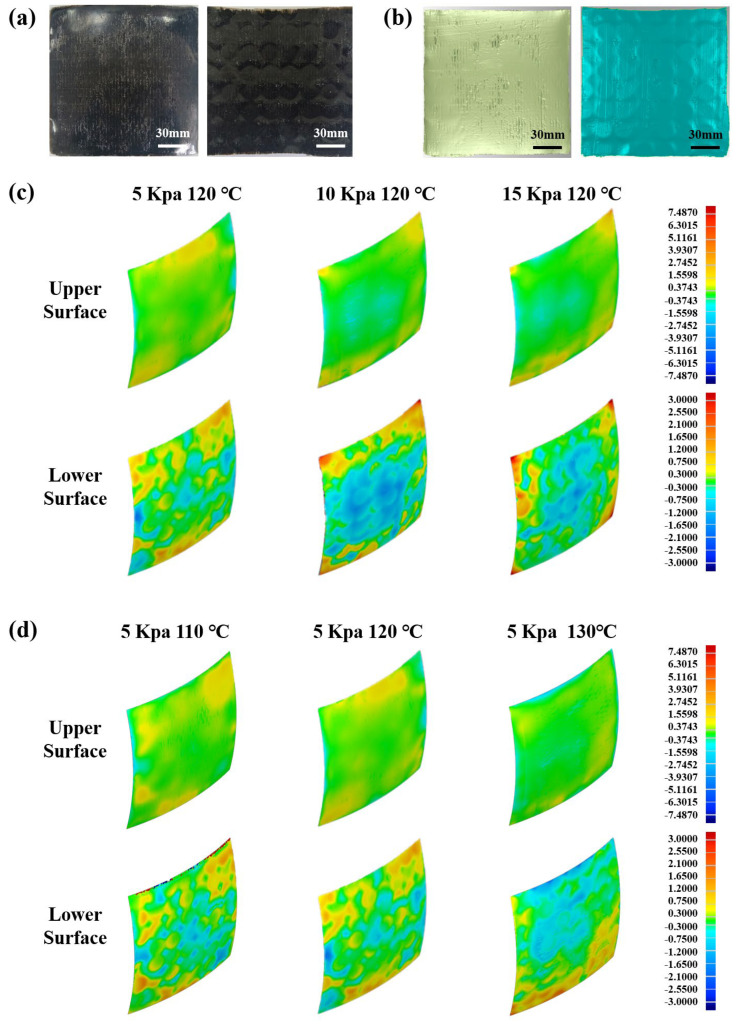
(**a**) The component and (**b**) the corresponding point cloud morphology produced by the GADF-MPFD process, and the geometrical errors of formed components under (**c**) different forming pressures and (**d**) different forming temperatures.

As the key process parameters in GADF-MPFD, pressure and temperature directly influence the formation of dimples. Figure 7a shows the physical appearance of the spherical composite component prepared by the GADF-MPFD process. Figure 7b shows the corresponding 3D point cloud morphology of the component in Figure 7a obtained by a portable optical 3D measuring instrument, which can accurately reflect the microscopic surface topography and geometrical shape of the component, and is the basis for the subsequent geometrical error analysis and profile extraction. The dimples were manifested as circular indentations upon the spherical head of the swinging pins. Since the lower surface of the sheets contacted the MPFD while the upper surface interfaced with the flexible silicone pad, dimples mainly appeared as unilateral depressions. By performing 3D comparisons between the surface fitted from point cloud data and the target surface, the geometrical errors of the components were obtained. The color scale limits on the lower surface were set to ±3 mm to visualize the depressions better. The geometrical errors of components under different pressures are shown in Figure 7c. As pressure increased, the dimples on the lower surface became significantly increased. For the upper surface, most areas were overall green. When the pressure was set as 10 kPa or 15 kPa, the blue area appeared at the center, and the yellow area at the edge was more prominent, indicating that the increase in pressure caused center compression and edge warping. The surface errors of components at different temperatures are shown in Figure 7d. The dimple conditions on the lower surface showed relatively minor differences. It was observed that, under various temperatures, the dimples were smaller in the central regions of the sheets but larger at the edge locations.

**Figure 8:**
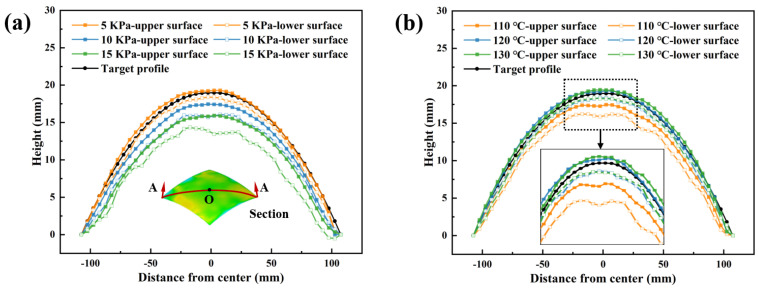
The diagonal profile under (**a**) different forming pressures and (**b**) different forming temperatures.

To further investigate the influence of process parameters on dimple formation in fabricated components, the diagonal profiles of both upper and lower surfaces were extracted. The diagonal of the profile of a 300 mm radius spherical surface was used as the reference for the target contour. Figure 8a presents the nodal variations along the diagonal under three different forming pressures. At a 5 kPa forming pressure, the extracted upper surface diagonal profile slightly exceeded the target surface contour. When pressure increased to 10 kPa, the component’s diagonal profile generally fell below the target contour. At 15 kPa, the overall profile showed significant deviation from the target spherical contour. The profile fluctuations of the lower surface intensified progressively with increasing pressure, and both the upper and lower surface contours gradually deviated from the target spherical surface. Figure 8b demonstrates that at a forming temperature of 110 °C, the lower surface profile exhibited significant deviation from the target contour, resulting in more severe dimple defects. When the forming temperature increased to 120 and 130 °C, the upper surface profiles of the formed components slightly exceeded the target contour while the lower surfaces remained marginally below it. Compared to the relatively smooth lower surface profiles observed at 120 and 130 °C, the 110 °C lower surface displayed considerably greater profile fluctuations. This temperature-dependent behavior suggested that 110 °C fell below the optimal processing window, leading to insufficient material flow and increased residual stresses, while temperatures of 120 °C and 130 °C provided better dimensional control.

**Figure 9:**
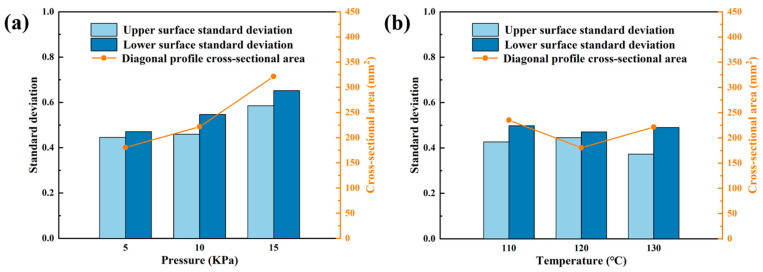
Standard deviation compared to the target profile and the diagonal profile cross-sectional area under (**a**) different forming pressures and (**b**) different forming temperatures.

The bar chart in Figure 9 illustrates the standard deviations between the component’s upper/lower surfaces and the target surface under different process parameters, while the line chart represents the cross-sectional areas enclosed by the upper and lower diagonal profiles. The standard deviation of the component’s lower surface served as a reliable indicator for assessing dimple defect severity. When forming pressures of 5, 10, and 15 kPa were applied, the corresponding standard deviations measured 0.4711, 0.5469, and 0.6523 mm, respectively. This progressive increase demonstrated that higher pressures generate greater contact forces between components and forming pins during processing, leading to more pronounced dimple defects. For the three forming temperatures evaluated, standard deviations of the component lower surfaces were 0.4982, 0.4711, and 0.4904 mm. Comparative analysis revealed that while temperature variations did influence dimple formation, their impact was significantly less substantial than that of forming pressure. The surface defects generated under the condition of 120 °C were the lightest. The cross-sectional area along the diagonal section was analyzed for quantitative evaluation of the dimple state. The results indicated a significant correlation between increasing forming pressure and expanding the intersection area. Excessive pressure compressed the resin into the area between the pins, thereby increasing the total cross-sectional area. Notably, components processed at both 110 and 130 °C consistently exhibited larger cross-sectional areas than those formed at the optimal 120 °C temperature, further confirming 120 °C as the most favorable thermal parameter for minimizing surface imperfections in this manufacturing process.

### 5.2. Effect of Interpolators on Dimple Defects

**Figure 10:**
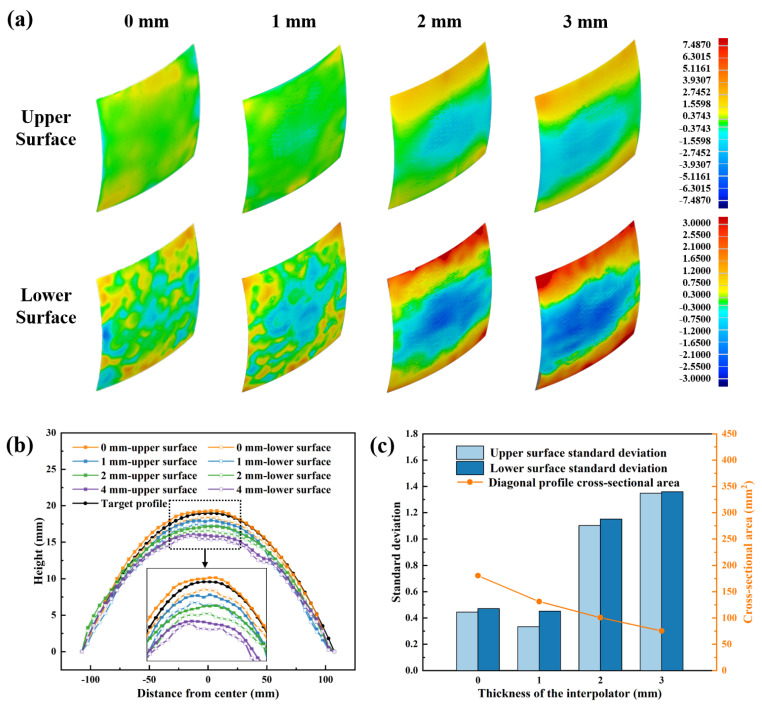
(**a**) Geometrical errors, (**b**) diagonal profile, and (**c**) standard deviation compared to the target profile and the diagonal profile cross-sectional area by using different thicknesses of interpolators.

While process optimization can reduce dimple defects to some extent, it cannot completely eliminate them. In this section, interpolators were used for dimple suppression in MPFD. As shown in Figure 10a, without an interpolator pad or with a 1 mm silicone interpolator, the upper surface of the formed sample appeared predominantly green in the contour map, while the lower surface exhibited mixed coloration, still revealing the circular dimple pattern. When an interpolator thickness of 2 mm or 3 mm was applied, the lower surface contour map became more uniform, with no discernible dimple shapes. However, the green area on the upper surface contour map decreased, indicating that the addition of silicone interpolators significantly affected the geometrical accuracy of the curved sheets.

Figure 10b presents the diagonal profiles of components with varying interpolator thicknesses. Without an interpolator or with a 1 mm interpolator, the upper and lower profiles exhibited substantial fluctuations and failed to closely match the target contour, particularly on the lower surface, which still displayed a wavy pattern. In contrast, with interpolator thicknesses of 2 and 3 mm, the upper and lower profiles align more closely, with only minor curvature variations on the lower surface. The standard deviation compared to the target profile and the diagonal profile cross-sectional area are summarized in Figure 10c. As the interpolator thickness increased, the cross-sectional area gradually decreased, confirming the effectiveness of thicker interpolators in suppressing dimples. However, when the interpolator thickness exceeded 2 mm, the standard deviation for both upper and lower surfaces increased sharply. The reduction in geometrical accuracy became the dominant factor influencing overall forming accuracy compared to dimple defects.

### 5.3. Analysis of Dimple Defects

**Figure 11:**
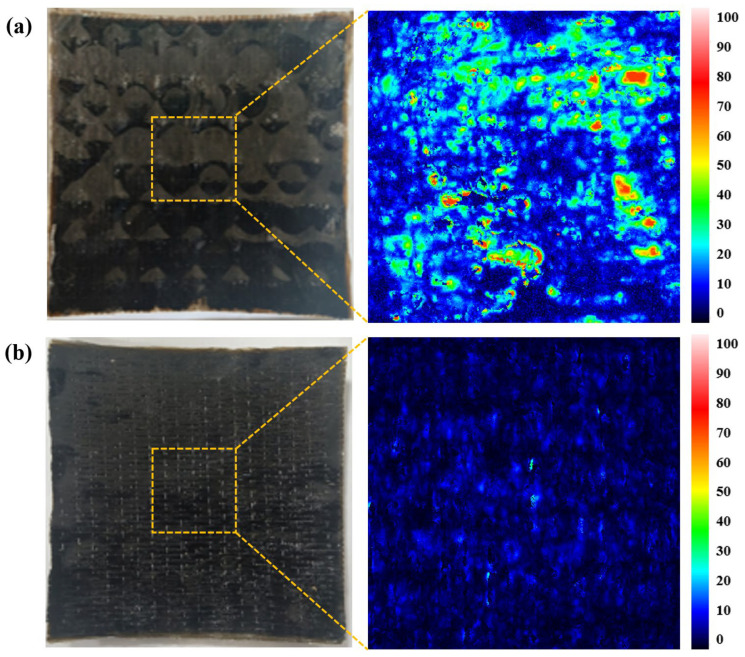
Ultrasonic C-scan results for (**a**) components with significant dimples and (**b**) dimple-free components.

The formation of dimple defects macroscopically affects the surface quality of components, while their internal impact on fiber-reinforced composite components remains unclear. Ultrasonic C-scan was employed to compare internal defects in the central sections of components with significant dimples and dimple-free components, as shown in Figure 11. The components with dimples exhibited numerous high-intensity zones whose shapes closely matched the circular dimple patterns. These findings indicate that the material contained substantial internal defects such as voids and delamination, with the dimple defects being the primary cause of these internal defects. In contrast, the dimple-free components displayed more uniform coloration in the resulting images, demonstrating that dimple suppression effectively reduces the formation of internal material defects.

**Figure 12:**
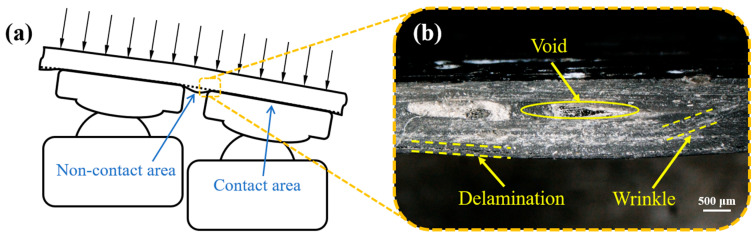
(**a**) A schematic diagram of dimple location and (**b**) microstructure of dimple cross-section.

Figure 12a illustrates the dimple formation mechanism schematically. The component’s lower surface could be divided into contact and non-contact areas, where the contact area provided reaction forces to counterbalance the top die pressure. In contrast, gaps between discrete pins in non-contact areas induce fiber wrinkling and resin accumulation. During curved sheet curing of fiber-reinforced composite prepregs, interlayer shear and interlayer slip created book-end effects to mitigate wrinkles [41]. As pressure increased, the normal pressure between fiber layers intensified, making interlayer slip more difficult. Fibers in direct contact with the MPFD would bend under pressure to form wrinkles, which further develop into dimple defects. On the other hand, when the forming temperature was too low, the higher viscosity resin increased the difficulty of interlayer slip, thereby raising the probability of wrinkle formation [42]. Conversely, when the forming temperature was too high, the deformation resistance of the fiber-reinforced composite decreased, making it more prone to wrinkle formation under the same pressure. Therefore, both excessively low and high temperatures could easily induce dimple defects. Figure 12b reveals the dimple’s cross-sectional microstructure. The brighter stripes in the cross-section of the component were fibers, while the darker areas were resin matrix. The unsupported non-contact area created gaps between curved lower fibers and nearly parallel upper fibers. Excessive fiber bending caused resin-filling failures and void defects. Vertical fiber reorientation within dimples generated internal wrinkling. While moderate wrinkling compensated for the insufficient global bending transition from non-contact to contact areas, severe fiber accumulation in deepest dimple regions induced interfacial delamination between bottommost and upper fiber layers.

The dimple defects are spatially confined to the non-contact areas between adjacent MPFD pins, and their lateral extent is consistently smaller than the area of the pin gap where they form. This observation confirms that dimple formation is a localized deformation phenomenon driven by stress concentration within the unsupported regions, rather than a global response to the applied forming pressure. From a process perspective, this relationship underscores that the elimination of dimples relies on mitigating stress gradients between the contact and non-contact zones, which validates the effectiveness of using a silicone interpolator to transition these stress states smoothly. Furthermore, this spatial correlation provides a design principle for future MPFD pin array optimization, where reducing the geometric discontinuity of the non-contact area would be a rational direction to minimize the occurrence and severity of dimple defects.

## 6. Geometrical Accuracy Compensation

**Figure 13:**
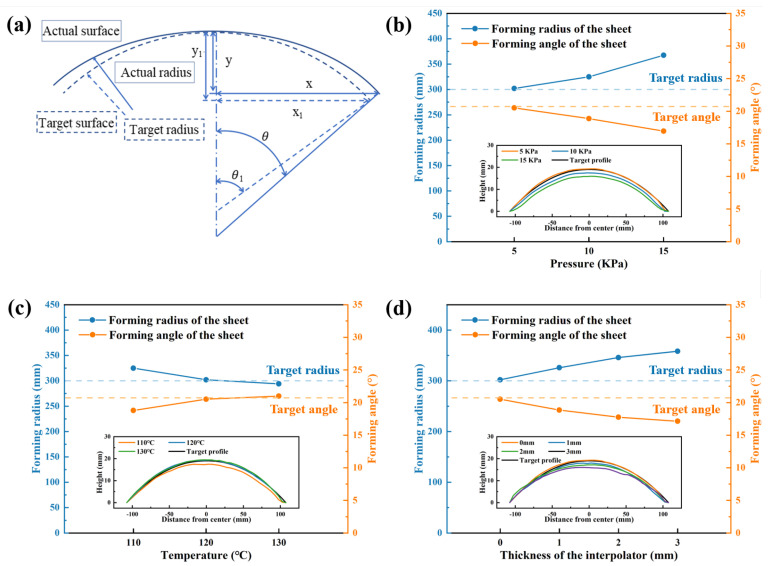
(**a**) A schematic diagram of the radius and angle of the component, and the effects of (**b**) pressure, (**c**) temperature, and (**d**) thickness of the interpolator on component geometrical accuracy.

Curing deformation, as an inherent defect, significantly impacts the forming accuracy of composite materials. Furthermore, since the components formed via GADF-MPFD require interpolators to suppress dimple defects, the introduction of these interpolators further reduces geometrical accuracy. Therefore, a characterization method based on forming radius and forming angle was proposed to evaluate the geometrical accuracy of fiber-reinforced composite spherical sheets, as illustrated in Figure 13a. The diagonal profile of the formed component was selected as the representative contour for calculations. The curing deformation angle can be defined as:

(8) $$\theta^{\prime }=\theta -\theta_{1}$$

where *θ* is the central angle corresponding to the actual formed curved surface, *θ*_1_ is the central angle corresponding to the target curved surface, and the calculation method of the central angle is as follows:

(9) $$\theta =\arcsin\frac{x}{R_{0}}$$

(10) $$\theta_{1}=\arcsin\frac{x_{1}}{R_{1}}$$

where *x* and *x*_1_ are half of the diagonal endpoints of the contours of the actual curved surface and the target curved surface, respectively, and *R*_0_ and *R*_1_ are the radius of the actual curved surface and the target curved surface, respectively. The radius of the actual curved surface can be calculated by the following formula:

(11) $${\left(R_{0}-y\right)}^{2}+x^{2}={R_{0}}^{2}$$

where *y* is the distance from the midpoint of the chord length to the contour of the formed curved surface.

### 6.1. Influence of Process Parameters and Interpolators on Geometrical Accuracy

The forming radius and forming angle of the upper surface were calculated to compare geometrical accuracy. Figure 13b illustrates the influence of forming pressure on component geometrical accuracy. As the forming pressure increased, the forming radius of the components expanded while the forming angle decreased. Since the forming pressure was maintained during the resin heating, melting and cooling stages, the resin flow was significantly affected by the changes in pressure. This resulted in pronounced fluctuations in the component profiles, with surface height decreasing as pressure increased. Typically, fiber-reinforced composites tend to exhibit inward-shrinkage curing deformation after forming [43]. However, in GADF-MPFD, under constant forming temperature, the increase in pressure effectively counteracted the reduction in the curvature radius caused by component shrinkage.

Figure 13c demonstrates that as temperature rose, the forming radius decreased while the forming angle increased. During the GADF-MPFD process, differential thermal expansion occurred between the composite material and the aluminum alloy die, with the resin’s coefficient of thermal expansion being substantially greater [44]. Consequently, despite maintaining forming pressure, significant resin shrinkage during cooling led to a reduced forming radius. At 110 °C, the forming radius exceeded the target value due to insufficient prepreg deformation caused by high resin viscosity. As forming temperature increased to 130 °C, the curing temperature gradient intensified, generating greater thermal stress and resulting in increased curing deformation and forming angles.

Figure 13d presents the impact of interpolator thickness on geometrical accuracy. Increasing the interpolator thickness progressively enlarged the forming radius. Thicker interpolators expanded the forming diameter. The elastic recovery trend of the interpolator under bending deformation generated a reaction force against the forming pressure, leading to the formation of errors.

After a comprehensive evaluation of dimple suppression and geometrical accuracy under various conditions, the optimal process parameters were determined as 5 kPa pressure, 120 °C temperature, and 2 mm interpolator.

### 6.2. Error Prediction and Surface Compensation

To further analyze the influence of component structural parameters on forming errors, the forming angles of experimental components in the CCD method were calculated, with results presented in Table 4. A quadratic regression model was established using the two-factor central composite design method with 11 experimental data points (Table 4) obtained from the CCD tests, expressed as:

(12) $$\begin{array}{l} R_{1}=17.21732-0.001689\times A-0.172323\times B+\\ 0.002127\times A\times B-0.003505\times A^{2}+0.000304\times B^{2} \end{array}$$

where *A* is the number of prepreg layers, and *B* is the target radius. The *p* value of the model is less than 0.0025, which is far less than the significance limit of 0.05, indicating that the model is highly significant. At the same time, the *p* value of the lack of fit item is 0.5547, which is greater than 0.05, indicating that the impact of the lack of fit item on the response value is not significant.

**Figure 14:**
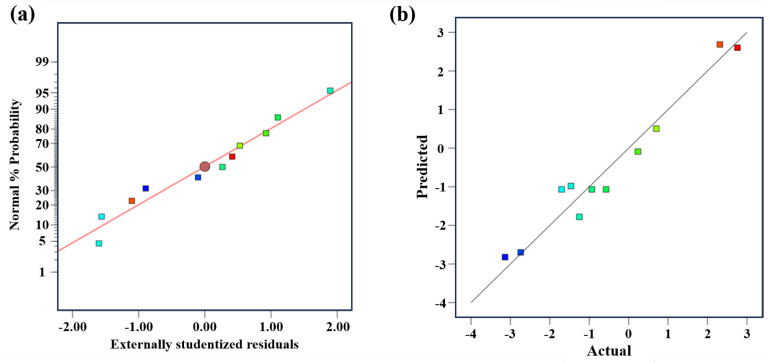
(**a**) Normal probability distribution of residuals, and (**b**) distribution of predicted and actual values.

The model’s validity was confirmed through the normal probability distribution plot of residuals and distribution of predicted and actual values, as shown in Figure 14. The 11 data points cover the full variable range of structural parameters, which ensures the model has high accuracy and generalization ability for the prediction of curing deformation angle within the research parameter range. In Figure 14a, the horizontal axis represents residual values while the vertical axis shows the percentage of normal distribution. The residual points of experimental data were distributed along a straight line, indicating the residuals followed a normal distribution. The coefficient of determination (R^2^) of this quadratic regression model is 0.987, which means 98.7% of the variation in the curing deformation angle can be explained by the structural parameters, verifying the excellent fitting degree and predictability of the established model. Therefore, the reliability of using the fitted quadratic polynomial to predict forming errors has been proven. Figure 14b plots the experimental values of curing deformation angle on the horizontal axis against the calculated values from the fitted model on the vertical axis. The experimental points were also distributed approximately along a straight line, confirming the good adaptability of the response surface methodology-fitted model.

**Figure 15:**
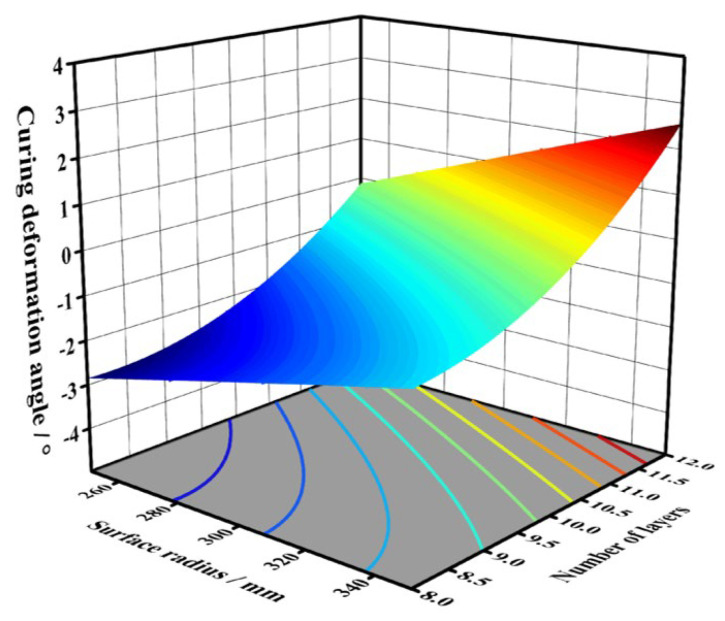
Response surface and contour diagram of the curing deformation angle.

Figure 15 presents the response surface and contour diagram of the curing deformation angle. The contour plot visually demonstrated the degree of the effects of structural parameters on experimental results. Under constant layers, as the target radius increased, the curing deformation angle of formed specimens showed an increasing trend. When the target radius remained constant, the curing deformation angle similarly increased with the additional number of layers. The response surface analysis revealed these nonlinear relationships between structural parameters and deformation characteristics, where both radius enlargement and layer addition contributed to progressively greater geometrical deviations from the target geometry.

The response surface model can be used to calculate deformation conditions for sheets with different numbers of layers and varying target surface radius. Based on the calculated deformation angles, the height of the pins in MPFD can be adjusted accordingly. Finally, the fabricated component that better conform to the target surface was obtained. To validate the model’s accuracy, experiments were conducted using a combination of nine layers of prepreg and a 270 mm target surface radius as structural parameters. Substituting these parameters into the model yielded a predicted curing deformation angle of −2.279°, while the actual measured curing deformation angle of the formed component was −2.856°, corresponding to a relative prediction error percentage of approximately 20.2%. Reverse calculation determined that an adjusted forming radius of 239 mm would compensate for this deformation.

**Figure 16:**
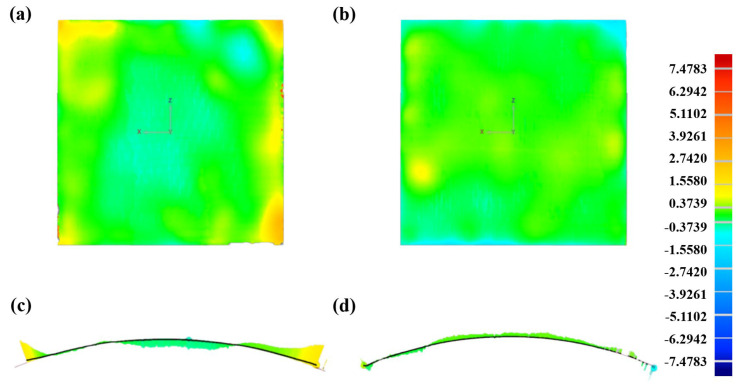
Geometrical errors of MPFD-reconfigured components: (**a**) original frontal profile, (**b**) reconfigured frontal profile, (**c**) original lateral profile, and (**d**) reconfigured lateral profile.

For experimental verification, two MPFDs were configured to construct spherical surfaces with a nominal radius of 270 mm and a compensated radius of 239 mm respectively. The forming process employed consistent parameters: a temperature of 120 °C, a pressure of 5 kPa, a 2 mm interpolator thickness, and nine prepreg layers. Figure 16 presents geometrical errors of the resulting components to demonstrate the effectiveness of the compensation method. By comparing Figure 16a,b, it was evident that the adjusted formed surface significantly improves its conformity with the target surface, while the application of interpolators effectively prevents the occurrence of dimple defects. The standard deviation value was used to quantitatively evaluate the degree of conformity between the formed surface and the target surface. The standard deviation of the component fabricated by the un-reconfigured MPFD was 0.5160 mm, which decreased to 0.3310 mm after compensation, representing a 38.85% reduction. The curing deformation angle was substantially reduced from −2.856° to 0.157°, with a deformation correction error percentage of only 5.5%, which further demonstrates the high practical application accuracy of the proposed prediction model. Figure 16c,d display the 2D cross-sectional comparison diagrams along the diagonal of the formed component, respectively. These clearly revealed that the edge regions of the sheet exhibited outward expansion of the surface profile, while the inner areas showed contraction. This expansion effect at both edges leads to contraction deformation in the central region. After the MPFD reconfiguration for surface compensation, the 2D cross-sectional comparison showed a significant reduction in surface deviation. Measured data indicated that the curing deformation angle before adjustment was −2.856°, which was close to the model-predicted value of −2.279°. After adjustment, the curing deformation angle was substantially reduced to 0.157°. These experimental results fully validate the high accuracy of the prediction model based on the RSM.

The proposed GADF-MPFD process exhibits notable advantages over traditional MPFD for composite forming. Traditional MPFD relies on rigid loading, which easily causes severe stress concentration of pin gaps and significant dimple defects [28,29]. In contrast, GADF-MPFD achieves flexible and uniform pressure transmission via the gas chamber and silicone pad, effectively mitigating stress concentration and suppressing dimples. Furthermore, unlike the single-variable control of traditional MPFD, the independent and precise control of pressure and temperature in GADF-MPFD, combined with surface compensation, has improved the forming geometrical accuracy by 38.85%.

## 7. Conclusions

In this research, the proposed new GADF-MPFD process and its mechanism for basalt fiber/epoxy resin composite sheets were demonstrated. The MPFD was employed to replace conventional solid dies, combined with the GADF method for fabricating composite curved sheets. Based on this concept, a comparative study was conducted to examine the effects of various process parameters and structural parameters on forming quality. The following conclusions were drawn:

(1) The GADF-MPFD system, equipped with a temperature controller and a pressure controller, enables the precise and effective control of forming temperature and pressure. By calculating pin heights through NURBS modeling and reconfiguring the pins, the MPFD achieves accurate controllability.
(2) Dimple defects primarily occur on the lower surface of components. The severity of the dimple increased with higher pressure, while temperature showed less impact. Under moderate temperature conditions, the surface accuracy of the component was relatively high. Increasing the thickness of interpolators effectively reduced dimples; however, excessive thickness led to diminished geometrical accuracy.
(3) Both pressure and interpolator pad thickness exhibited proportional relationships with forming radius and increasing these parameters gradually reduced component geometrical accuracy. A moderate temperature was proven to be optimal for geometrical accuracy. As the temperature rose, component shapes first approached and then deviated from the target surface.
(4) Considering both dimple suppression and geometrical accuracy, the optimal process parameters were determined as 5 kPa pressure, 120 °C temperature, and a 2 mm interpolator.
(5) The influence of two structural parameters, prepreg layer number and target radius, on forming accuracy was analyzed using RSM, and the curing deformation of the component was predicted. Increases in both the number of layers and the target radius resulted in larger curing deformation angles. The fitted model was validated using spherical components with a 270 mm target radius. Components formed by the model-modified MPFD showed 38.85% improved accuracy compared to unmodified MPFD-formed components.

## Figures and Tables

**Table 1:** Temperature test results of thermal images at different target heating conditions.

Test Area	Maximum Temperature (°C)	Minimum Temperature (°C)	Average Temperature (°C)
R1	111.3	99.0	106.8
L1	110.3	99.7	106.1
R2	120.2	110.3	116.0
L2	119.9	115.5	115.8
R3	130.4	120.0	126.0
L3	130.1	121.8	125.9

**Table 2:** Experimental plan for GADF-MPFD.

Test Number	Pressure (kPa)	Temperature (°C)	Interpolator Thickness (mm)
A1	5	120	0
A2	10	120	0
A3	15	120	0
A4	5	110	0
A5	5	130	0
A6	5	120	1
A7	5	120	2
A8	5	120	3

**Table 3:** Experimental design plan of CCD.

Test Number	Factor 1: Number of Layers	Factor 2: Surface Radius (mm)
B1	8	250
B2	12	250
B3	8	350
B4	12	350
B5	7	300
B6	13	300
B7	10	229
B8	10	371
B9	10	300
B10	10	300
B11	10	300

**Table 4:** Experimental results of CCD.

Test Number	Forming Angle (°)
B1	−3.136
B2	−1.463
B3	0.235
B4	2.759
B5	−2.739
B6	0.702
B7	−1.251
B8	2.314
B9	−0.934
B10	−1.701
B11	−0.573

## Data Availability

The original contributions presented in this study are included in the article. Further inquiries can be directed to the corresponding author.
